# The effect of Neuroligin-2 absence on sleep architecture and electroencephalographic activity in mice

**DOI:** 10.1186/s13041-018-0394-3

**Published:** 2018-09-19

**Authors:** Bong Soo Seok, Erika Bélanger-Nelson, Chloé Provost, Steve Gibbs, Valérie Mongrain

**Affiliations:** 1 Research Center and Center for Advanced Research in Sleep Medicine, Hôpital du Sacré-Cœur de Montréal (CIUSSS-NIM), 5400 Gouin West blvd, Montréal, QC H4J 1C5 Canada; 2 Department of Neuroscience, Université de Montréal, 2960 chemin de la Tour, Montreal, QC H3T 1J4 Canada

**Keywords:** Neuroligin, Cell adhesion molecule, Knockout mice, Electroencephalography, Sleep regulation, Wakefulness, Delta activity

## Abstract

Sleep disorders are comorbid with most psychiatric disorders, but the link between these is not well understood. Neuroligin-2 (NLGN2) is a cell adhesion molecule that plays roles in synapse formation and neurotransmission. Moreover, NLGN2 has been associated with psychiatric disorders, but its implication in sleep remains underexplored. In the present study, the effect of *Nlgn2* knockout (*Nlgn2*^*−/−*^) on sleep architecture and electroencephalographic (EEG) activity in mice has been investigated. The EEG and electromyogram (EMG) were recorded in *Nlgn2*^*−/−*^ mice and littermates for 24 h from which three vigilance states (i.e., wakefulness, rapid eye movement [REM] sleep, non-REM [NREM] sleep) were visually identified. Spectral analysis of the EEG was performed for the three states. *Nlgn2*^*−/−*^ mice showed more wakefulness and less NREM and REM sleep compared to wild-type (*Nlgn2*^*+/+*^) mice, especially during the dark period. This was accompanied by changes in the number and duration of individual episodes of wakefulness and sleep, indexing changes in state consolidation, as well as widespread changes in EEG spectral activity in all states. Abnormal ‘hypersynchronized’ EEG events have also been observed predominantly in *Nlgn2*^*−/−*^ mice. These events were mainly observed during wakefulness and REM sleep. In addition, *Nlgn2*^*−/−*^ mice showed alterations in the daily time course of NREM sleep delta (1–4 Hz) activity, pointing to modifications in the dynamics of sleep homeostasis. These data suggest that NLGN2 participates in the regulation of sleep duration as well as EEG activity during wakefulness and sleep.

## Introduction

Research from the last decades suggests that people suffering from a sleep disorder such as insomnia show higher risk of developing medical and/or psychiatric disorders [1, 2]. On the other hand, most psychiatric disorders are associated with sleep disturbances [3]. For instance, decreased sleep efficiency and consolidation is characterizing autism spectrum disorders (ASDs) and schizophrenia [3]. However, the molecular links between sleep alterations and comorbid disorders remain poorly understood. Neurodevelopmental psychiatric disorders in particular, such as ASDs and schizophrenia, have been proposed to originate from changes in the relative balance of excitation to inhibition (E/I) in the central nervous system. For instance, indications of reduced γ-aminobutyric acid (GABA) ergic tone can result in an increased E/I ratio in ASDs [4], while *N*-methyl-D-aspartate (NMDA) receptor hypofunction likely also modifies the E/I ratio in schizophrenia [5]. Neuroligins (NLGNs) are postsynaptic adhesion proteins that have been shown to regulate synaptogenesis, synaptic function, and E/I balance [6, 7]. Importantly, mutations in *Nlgn* genes have been linked to the abovementioned neuropsychiatric disorders [6], such as missense mutations of *Nlgn2* in schizophrenia [8]. Therefore, understanding the roles of NLGNs in the regulation of sleep quantity and quality could help to identify mechanisms underlying the relationship between neuropsychiatric disorders and sleep disturbances.

Research in both rodents and flies has indeed provided support for an implication of specific NLGNs in sleep regulation [9]. In mice, the absence of NLGN1 (knockout, KO) results in a decreased duration of wakefulness and increased duration of NREM sleep accompanied by changes in wakefulness and NREM sleep quality as quantified using EEG spectral analysis and slow wave detection [10, 11]. In rats, the absence of NLGN3 (KO) was also recently shown to impact sleep duration and quality, and more precisely to result in decreased NREM sleep duration and increased REM sleep duration, and in multiple modifications in EEG activity in all states [12]. *Nlgn3*^*R451C*^ knock-in mice, carrying an ASD-associated missence mutation, have been shown to exhibit normal sleep architecture but a decrease in low frequency (< 10 Hertz [Hz]) activity during NREM sleep [13]. To our knowledge, no data is available yet regarding the sleep phenotype in rodents with genetic manipulation of *Nlgn2* or *Nlgn4*.

NLGN2 is preferentially localized at inhibitory synapses [7, 14]. It regulates inhibitory synaptic transmission, which has been unveiled through manipulations of *Nlgn2* expression level. *Nlgn2* overexpression in mice increased the frequency of miniature inhibitory postsynaptic currents (mIPSCs) in pyramidal cells of the prefrontal cortex compared to control mice [15]. On the other hand, in the absence of NLGN2, both the amplitude and frequency of mIPSCs are decreased in the mouse medial prefrontal cortex [16] and the amplitude of mIPSCs is decreased in granule cells of the dentate gyrus of the hippocampus [17]. These modulations of NLGN2 level did not affect miniature excitatory postsynaptic currents [15, 16], thus modifying the E/I balance [17]. Of importance is that NLGN2 was shown to impact inhibition in a cell type-specific manner within the same circuit (i.e., neocortex) [18]. Inhibitory/GABAergic transmission has been implicated in sleep regulation, notably in the context of the flip-flop switch model of sleep regulation [19], and in that of the development of hypnotic drugs for insomnia treatment [20]. Indeed, the flip-flop switch model suggests that GABAergic sleep-promoting regions are inhibiting monoaminergic wake-promoting regions to control vigilance state transitions [19], whereas hypnotic drugs such as benzodiazepines are GABA_A_ receptor agonists promoting light NREM sleep [21, 22]. In the absence of NLGN2, the decrease in mIPSC could result in weakening of sleep-promoting regions and to opposite effects than GABA_A_ receptor agonists, therefore to more wakefulness and less sleep. Given the role of GABA_A_ receptors in shaping EEG activity [20], and the results showing impaired mechanisms linked to the clustering of GABA_A_ receptors at synaptic sites in *Nlgn2*^*−/−*^ mice [23], alterations in EEG activity are also expected in absence of NLGN2.

The general objective of this study was to evaluate the role of NLGN2 in wakefulness and sleep regulation. EEG-EMG recordings have been used to assess changes in normal/undisturbed wake/sleep architecture as well as EEG activity in mice lacking NLGN2 (*Nlgn2*^*−/−*^ mice) in comparison with wild-type (*Nlgn2*^*+/+*^) and heterozygous (*Nlgn2*^*+/−*^) littermates. Results are indicative of changes in both wake/sleep quantity and quality in *Nlgn2*^*−/−*^ mice, and reveal a state-dependent occurrence of abnormal EEG events. Our findings thus suggest that NLGN2, in addition to NLGN1 and NLGN3, modulates both the architecture and quality of wakefulness and sleep, which will help to understand mechanisms underlying comorbidity between brain diseases and sleep disorders.

## Methods

### Animals

Mixed genetic background (B6;129-*Nlgn2*^*tm1Bros*^/J) mice were purchased from Jackson Laboratories and bred on site by placing one male and one female *Nlgn2*^*+/−*^ mice in a breeding cage to generate three genotypes: homozygous *Nlgn2*^*−/−*^ mice, and *Nlgn2*^*+/−*^ and *Nlgn2*^*+/+*^ littermates. *Nlgn2*^*−/−*^ mice were previously generated by homologous recombination [24]. Briefly, a targeting vector containing a neomycin resistance cassette was electroporated into 129Sv-derived embryonic stem cells to disrupt the sequence of the first *Nlgn2* exon covering the translational start site and 380 base-pair of the 5′ coding sequence, and recombinant stem cells were transfected into C57BL/6 blastocysts.

Male mice only were used in our study to reduce variability due to sex because sex differences in sleep architecture and EEG activity have been reported [25, 26]. We studied 14 *Nlgn2*^*+/+*^ mice, 14 *Nlgn2*^*+/−*^ mice, and 12 *Nlgn2*^*−/−*^ mice. At the time of the surgery (see below), *Nlgn2*^*+/+*^ mice were 69.2 ± 2 days old (range 57 to 83 days) and weighing 27.5 ± 0.6 g; *Nlgn2*^*+/−*^ mice were 71.8 ± 1.9 days old (range 57 to 83 days) and 27.3 ± 0.8 g; and *Nlgn2*^*−/−*^ mice were 70.8 ± 2.0 days old (range 58 to 83 days) and 24.3 ± 0.8 g. Weight was significantly different between genotypes (F_2,37_ = 6.3, *p* = 0.004). *Nlgn2*^*−/−*^ mice were significantly lighter than both *Nlgn2*^*+/+*^ and *Nlgn2*^*+/−*^ mice (*p* = 0.003 and *p* = 0.005, respectively) while there was no significant difference between *Nlgn2*^*+/+*^ and *Nlgn2*^*+/−*^ mice (*p* = 0.8).

### Electrode implantation surgery

The surgery of electrode implantation for recording the EEG and EMG was performed as detailed previously [10, 27]. Briefly, when mice were between 9 and 10 weeks of age, EEG/EMG implantation surgery was performed under deep Ketamine/Xylazine anesthesia (120/10 mg/kg, intraperitoneal injection). Mice were placed in a stereotaxic frame and two gold-plated screws (diameter 1.1 mm), which served as EEG electrodes, were screwed through the skull over the right cerebral hemisphere (anterior: 1.7 mm lateral to midline, 1.5 mm anterior to bregma; posterior: 1.7 mm lateral to midline, 1.0 mm anterior to lambda). An additional screw, which served as a reference, was implanted on the right hemisphere (2.6 mm lateral to midline, 0.7 mm posterior to bregma). Three anchor screws were implanted on the left hemisphere. Two gold wires serving as EMG electrodes were inserted between neck muscles. EEG and EMG electrodes were soldered to a connector and secured on the skull with cement. After four days of recovery, mice were connected to a swivel contact and habituated to the cabling condition for a week before recording.

### Protocol and EEG recording

Starting 2 weeks before surgery and throughout the experiment, mice were housed in individual cages and kept under a 12-h light/12-h dark cycle at a temperature between 23 and 25 °C, with free access to food and water. Electrophysiological signals were continuously recorded for 24 h starting at light onset (Zeitgeber time 0: ZT0). Signals were amplified (Lamont amplifiers), sampled at 256 Hz, and filtered using the software Stellate Harmonie (Natus, San Carlos, CA). A bipolar montage was subsequently employed for identification of vigilance states and spectral analysis (signal used representing the difference between the anterior and the posterior EEG electrodes).

### EEG analyses

The sampled signals were segmented in 4-s epochs to visually identify vigilance states (wakefulness, NREM sleep, REM sleep) based on EEG and EMG features characterizing the different vigilance states as previously described [28]. During state identification, artifacts such as abnormal peaks and abnormal mixture of frequencies, and transitions were identified and excluded from subsequent EEG spectral analysis (see below). Parameters related to wakefulness and sleep duration/architecture as well as to wakefulness and sleep consolidation/fragmentation were directly calculated from vigilance state identification. More precisely, the time spent in vigilance states (in min) was calculated for the full 24-h, the first 12-h (Light period), the second 12-h (Dark period) and per hour. Mean duration of individual episodes of vigilance states (in sec) and total number of individual vigilance state episodes during the 12-h Light and the 12-h Dark have also been computed.

Spectral analysis was performed to investigate EEG activity during the different vigilance states (as a measure of state quality). The bipolar EEG signal was decomposed into its constituent frequency components using fast Fourier transform. The EEG power density was calculated between 1 and 50 Hz (1-Hz resolution) during wakefulness, NREM sleep and REM sleep for the full 24-h. In addition to absolute power spectra computed as performed previously [10], relative power density was also computed for which the activity of each Hz-bin was expressed relative to the mean power density of all bins of all states of the mouse as previously done [13, 27]. Absolute power spectra can quantify genotype differences in vigilance state quality while providing information regarding the cytoarchitecture of the cerebral cortex, whereas, relative power spectra removes the variability of signal due to, for instance, general alterations in cortical structure and/or differences in the depth of EEG electrodes, and can allow for more accurate observations of vigilance state-specific genotype differences.

The time course of NREM sleep EEG delta (between 1 and 4 Hz) activity was computed to analyze the dynamics of the sleep homeostat [29, 30]. The change in absolute delta power was monitored throughout 18 intervals over the 24-h (12 intervals comprising the same number of epochs of NREM sleep during the light phase and 6 intervals with the same number of NREM sleep epochs in the dark phase) similar to previously performed [10]. Relative delta power dynamics was also computed with respect to the 24-h mean delta power of each animal, which allow to better highlight delta activity dynamics by removing intra-individual differences in absolute activity [27, 28]. The time course of theta (6–9 Hz) activity during wakefulness was also analyzed in absolute and relative values for 18 intervals comprising 6 equal intervals during the 12-h Light and 12 equal intervals during the 12-h Dark to take into account the 24-h distribution of wakefulness. For this last analysis, one *Nlgn2*^*+/+*^ mouse was not included because of an absence of wakefulness for one interval.

### Abnormal EEG event identification

During vigilance state identification, occasional and distinct events of high amplitude EEG bursts were observed. Since alteration in E/I balance has been postulated as a mechanism underlying epileptogenesis and seizure generation [31, 32], this abnormal EEG activity might be indicative of hypersynchronisation and/or epileptiform activity. Thus, the abnormal EEG events were marked on EEG traces (and excluded from the spectral analysis of the vigilance states described above). More precisely, the number and duration of abnormal events were quantified by marking them according to the following two criteria: amplitude at least twice that of the background EEG signal, and duration of at least one second. Two events separated by less than 0.5 s were considered as a single one. The EEG power density was calculated between 1 and 50 Hz (1-Hz resolution) for abnormal events, from which the peak frequency was determined for each mouse.

### Statistical analyses

Vigilance state variables and event features calculated for the 24-h, 12-h Light and 12-h Dark were compared between genotypes using one-way analyses of variance (ANOVAs). Vigilance state variables with significant genotype difference were decomposed using Tukey post hoc or planned comparisons. Vigilance state variables calculated per hour and time course of delta and theta activity per intervals were analyzed using two-way repeated-measure ANOVAs. Significance level for repeated-measure ANOVAs was adjusted by Huynh-Feldt correction and significant differences were decomposed by planned comparisons. Finally, comparison of the number of events between the 12-h Light and 12-h Dark as well as between states in *Nlgn2*^*−/−*^ mice was performed using one-way repeated-measure ANOVAs. Data are reported as mean and standard error of the mean (SEM), and the threshold for statistical significance was set to 0.05.

## Results

### More time spent awake in *Nlgn2*^*−/−*^ mice

**Fig. 1:**
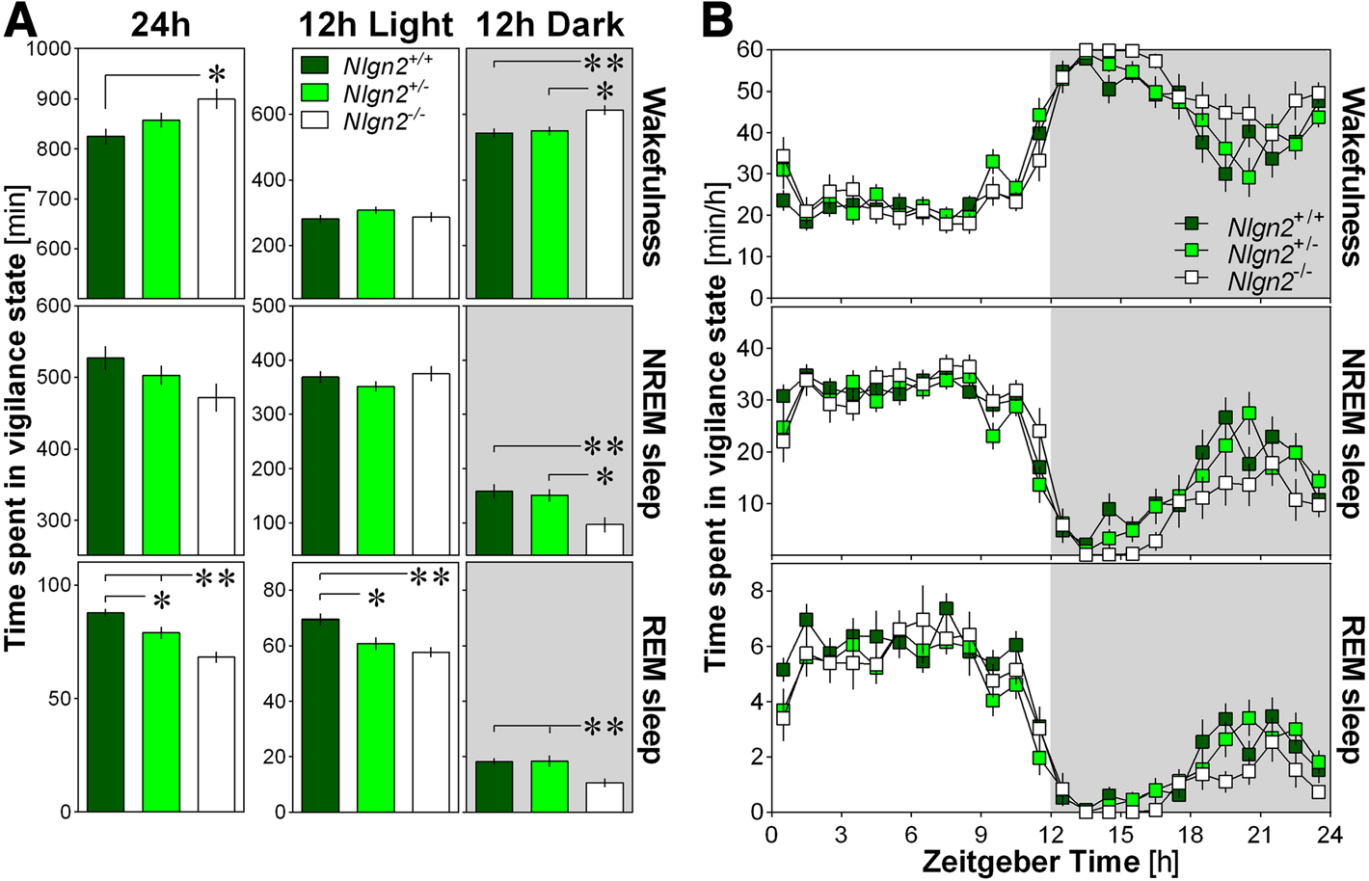
Time spent (min ± SEM) in wakefulness, NREM sleep and REM sleep in *Nlgn2*^*+/+*^, *Nlgn2*^*+/−*^ and *Nlgn2*^*−/−*^ mice measured using EEG and EMG recordings. **a**) Total time spent in wake, NREM sleep and REM sleep for the total 24 h recording, the 12 h Light and the 12 h Dark periods. Significant genotype effects were found for 24 h wake (F_2,37_ = 4.8, *p* = 0.014), 24 h REM sleep (F_2,37_ = 16.9, *p* < 0.0001), 12 h Light REM sleep (F_2,37_ = 8.5, *p* < 0.001), 12 h Dark wake (F_2,37_ = 6.8, *p* = 0.003), 12 h Dark NREM sleep (F_2,37_ = 6.4, *p* = 0.004) and 12 h Dark REM sleep (F_2,37_ = 6.4, p = 0.004). No significant genotype effect was found for 24 h NREM sleep (F_2,37_ = 2.7, *p* = 0.08), 12 h Light wake (F_2,37_ = 1.3, *p* = 0.3) and 12 h Light NREM sleep (F_2,37_ = 1.1, p = 0.3). Stars show significant post-hoc Tukey HSD comparisons between indicated genotypes (*: *p* < 0.05; **: *p* < 0.01). **b**) Hourly distribution of wake, NREM sleep and REM sleep. For wakefulness, significant genotype and time effects were found (respectively, F_2,37_ = 4.8, p = 0.014 and F_23,851_ = 51.3, *p* < 0.0001), but no significant interaction was observed (F_46,851_ = 1.2, *p* = 0.14). For NREM sleep, a significant time effect was found (F_23,851_ = 47.0, p < 0.0001), but no significant genotype effect or interaction was observed (respectively, F_2,37_ = 2.7, p = 0.08 and F_46,851_ = 1.3, *p* = 0.07). For REM sleep, significant genotype and time effects were found (respectively, F_2,37_ = 16.9, p < 0.0001 and F_23,851_ = 52.3, p < 0.0001), but no significant interaction was observed (F_46,851_ = 0.8, *p* = 0.7). Grey backgrounds indicate the 12 h dark period

During the full 24-h recording, *Nlgn2*^*−/−*^ mice spent more time in wakefulness and less time in REM sleep compared to *Nlgn2*^*+/+*^ littermates (Fig. 1a). A significant difference in REM sleep amount was also observed between *Nlgn2*^*+/−*^ mice and the other two genotypes, indicating a gene dosage-dependent effect. The increased time spent in wakefulness in *Nlgn2*^*−/−*^ mice originated from a change specific to the 12-h Dark period during which significant differences were observed in comparison to both *Nlgn2*^*+/−*^ and *Nlgn2*^*+/+*^ littermates (Fig. 1a). Also specifically for the 12-h Dark period, *Nlgn2*^*−/−*^ mice showed significantly less NREM sleep than *Nlgn2*^*+/−*^ and *Nlgn2*^*+/+*^ mice (Fig. 1a). Less REM sleep in both *Nlgn2*^*−/−*^ and *Nlgn2*^*+/−*^ mice compared to *Nlgn2*^*+/+*^ mice was observed for the 12-h Light period, while less REM sleep in *Nlgn2*^*−/−*^ mice compared to *Nlgn2*^*+/−*^ and *Nlgn2*^*+/+*^ mice was found for the 12-h Dark period (Fig. 1a). These observations suggest impairment in mechanisms generating sleep in the absence of NLGN2.

The 24-h distribution of wakefulness and sleep states in *Nlgn2*^*−/−*^ mice was further analyzed using hourly values (Fig. 1b). The typical predominance of sleep during the light period and of wakefulness during the dark period was equally observed in all three genotypes. Moreover, the distribution was not significantly affected by genotype for all three vigilance states indicating a dispersed effect of the mutation that accumulates over many hours to result in the changes in wakefulness and sleep amount described above.

### Increased consolidation of vigilance states in *Nlgn2*^*−/−*^ mice

**Fig. 2:**
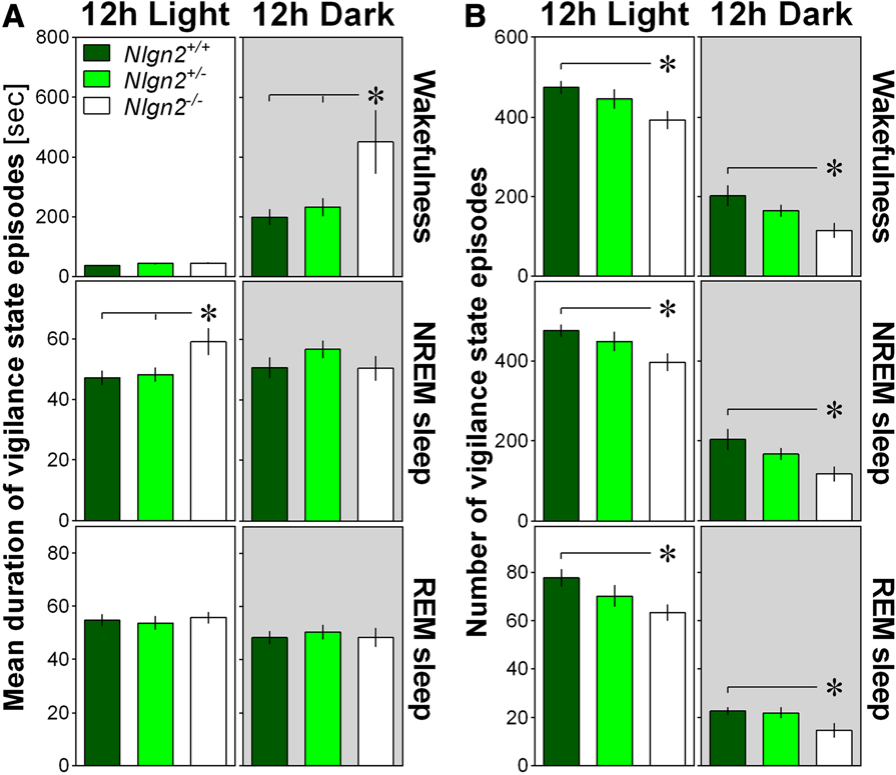
Parameters of vigilance state consolidation/fragmentation in *Nlgn2*^*+/+*^, *Nlgn2*^*+/−*^ and *Nlgn2*^*−/−*^ mice quantified for the 12 h Light and the 12 h Dark periods using EEG and EMG recordings. **a**) Mean duration of individual episodes of vigilance states. Significant genotype effect was found for 12 h Light NREM sleep (F_2,37_ = 4.3, *p* = 0.02) and 12 h Dark wake (F_2,37_ = 4.9, p = 0.01). No significant genotype effect was found for 12 h Light wake (F_2,37_ = 3.2, *p* = 0.052), 12 h Light REM sleep (F_2,37_ = 0.2, *p* = 0.8), 12 h Dark NREM sleep (F_2,37_ = 1.1, p = 0.3), and 12 h Dark REM sleep (F_2,37_ = 0.1, *p* = 0.9). **b**) Number of individual episodes of vigilance states. Significant genotype effects were observed for all states for both the 12 h Light and 12 h Dark (12 h Light wake F_2,37_ = 3.6, *p* = 0.04; 12 h Light NREM sleep F_2,37_ = 3.5, p = 0.04; 12 h Light REM sleep F_2,37_ = 3.3, *p* = 0.048; 12 h Dark wake F_2,37_ = 4.3, p = 0.02; 12 h Dark NREM sleep F_2,37_ = 4.2, p = 0.02; 12 h Dark REM sleep F_2,37_ = 3.5, p = 0.04). Stars show significant post-hoc Tukey HSD comparisons between indicated genotypes (*: *p* < 0.05; **: *p* < 0.01). Grey backgrounds indicate the 12 h dark period

The mean duration of individual episodes of vigilance states and the total number of individual episodes of each state were then calculated in *Nlgn2*^*−/−*^ mice separately for the 12-h Light and Dark periods to better understand the origin of alterations in vigilance state duration. Moreover, these variables are indicative of wakefulness and sleep consolidation/fragmentation. The mean duration of individual episodes of NREM sleep was longer in *Nlgn2*^*−/−*^ mice than in the two other genotypes during the 12-h Light period, and the same finding emerged regarding the mean duration of individual wakefulness episodes during the 12-h Dark period (Fig. 2a). Concerning the number of individual episodes, it was lower in *Nlgn2*^*−/−*^ mice than in *Nlgn2*^*+/+*^mice only for all three vigilance states and for both the 12-h Light and Dark periods (Fig. 2b). In sum, longer and fewer episodes of the different vigilance states suggest increased wakefulness and sleep consolidation in the absence of NLGN2.

### Abnormal EEG events in *Nlgn2*^*−/−*^ mice

**Fig. 3:**
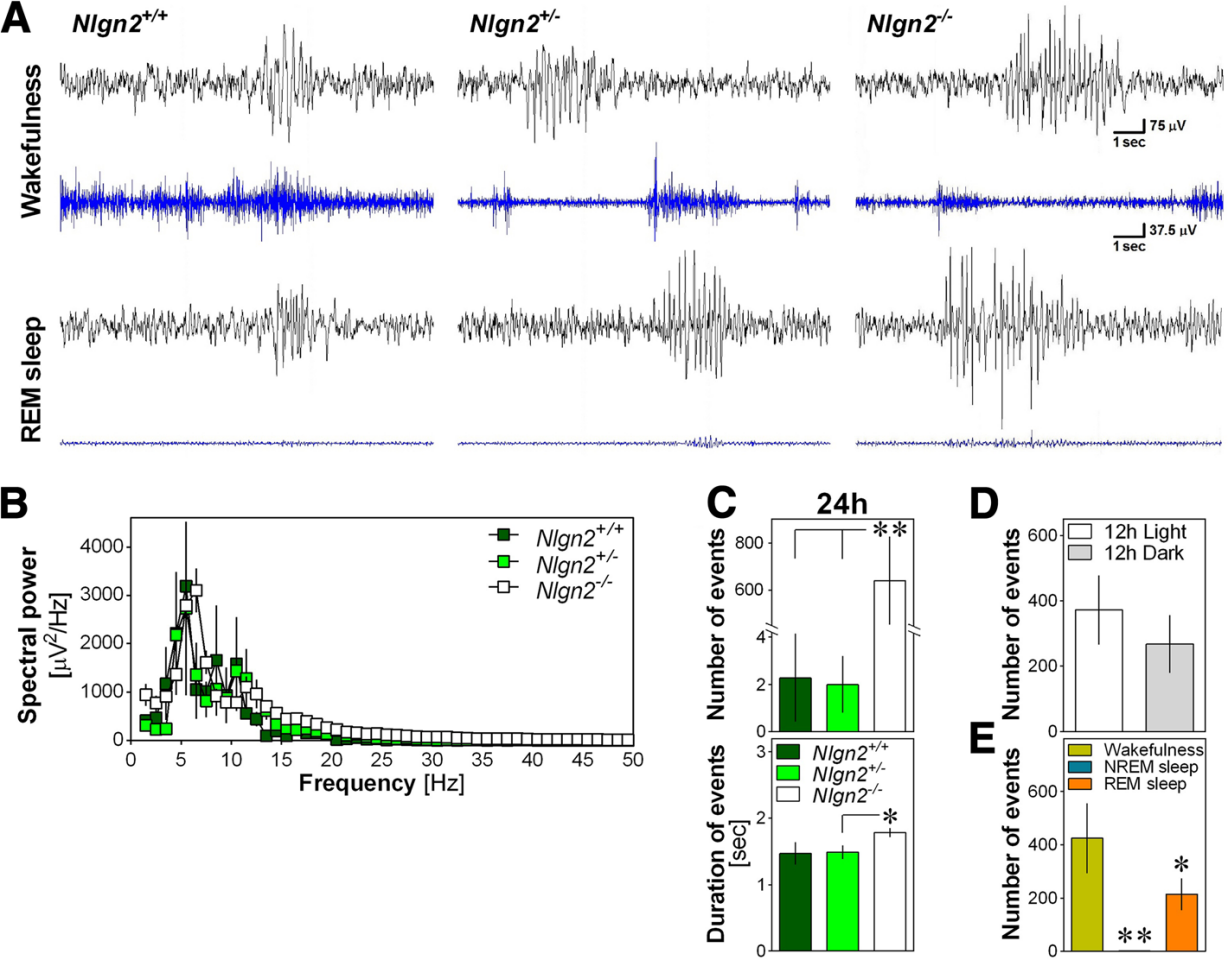
Abnormal EEG events in *Nlgn2*^*+/+*^, *Nlgn2*^*+/−*^ and *Nlgn2*^*−/−*^ mice. **a**) Representative 12-s EEG traces (black), and corresponding EMG traces (blue), showing abnormal EEG events for mice of the three genotypes during wakefulness (upper traces) and REM sleep (lower traces). Scale bars are the same for the two states of all mice. **b**) Absolute spectral power of events calculated between 1 and 50 Hz and averaged for each genotype for mice showing events (*n* = 3 *Nlgn2*^*+/+*^, *n* = 5 *Nlgn2*^*+/−*^, *n* = 12 *Nlgn2*^*−/−*^). **c**) Total number of events (upper panel) observed for the 24 h recording in the three genotypes, and their mean duration for mice showing events (lower panel). The number of events was significantly affected by genotype (F_2,37_ = 13.7, *p* < 0.0001). **: p < 0.01 between indicated points (planned comparisons). For mice showing events, the duration of events was significantly affected by genotype (F_2,17_ = 3.7, *p* < 0.05). *: p < 0.05 between indicated points (planned comparisons). **d**) Number of events calculated separately for the 12 h Light and the 12 h Dark periods in *Nlgn2*^*−/−*^ mice only. The number of events did not significantly differ between the 12 h Light and the 12 h Dark periods (F_1,11_ = 3.5, *p* = 0.09). **e**) Number of events calculated separately for the three vigilance states in *Nlgn2*^*−/−*^ mice only. The number of events was significantly different between vigilance states (F_2,22_ = 9.9, *p* < 0.001). **: *p* < 0.01 compared to the other two states; *: p < 0.05 compared to wakefulness

In the course of EEG visual inspection, abnormal bursts of high amplitude ‘hypersynchronized’ EEG activity were observed predominantly in *Nlgn2*^*−/−*^ animals and in wakefulness and REM sleep (Fig. 3a). These abnormal events were observed in 3 out of 14 *Nlgn2*^*+/+*^ mice (21.4% of the group; between 1 and 26 events per mouse), in 5 out of 14 *Nlgn2*^*+/−*^ mice (35.7%; between 1 and 16 events per mouse), and in all 12 *Nlgn2*^*−/−*^ mice (between 1 and 2116 events per mouse). Spectral analysis of these events revealed a high peak in the theta range (i.e., 4–8 Hz; Fig. 3b), with an average peak frequency of 6.5 ± 1.0 Hz in *Nlgn2*^*+/+*^ mice, 7.7 ± 1.4 Hz in *Nlgn2*^*+/−*^ mice, and 6.2 ± 0.1 Hz in *Nlgn2*^*−/−*^ mice. The average peak frequency was not significantly affected by genotype (F_2,17_ = 1.4, *p* > 0.2), but *Nlgn2*^*−/−*^ mice showed significantly more events than the other two genotypes (Fig. 3c). The duration of these abnormal events was also significantly affected by genotype with *Nlgn2*^*−/−*^ mice showing longer events than *Nlgn2*^*+/−*^ mice (Fig. 3c). In *Nlgn2*^*−/−*^ mice, there was no difference in the number of observed events between the 12-h Light and the 12-h Dark periods (Fig. 3d), but events were significantly more frequent during wakefulness than during REM sleep, and very rare during NREM sleep (Fig. 3e). Therefore, the quality of the EEG during both wakefulness and REM sleep seems to be particularly affected by the absence of NLGN2.

### Widespread changes in EEG activity in *Nlgn2*^*−/−*^ mice

**Fig. 4:**
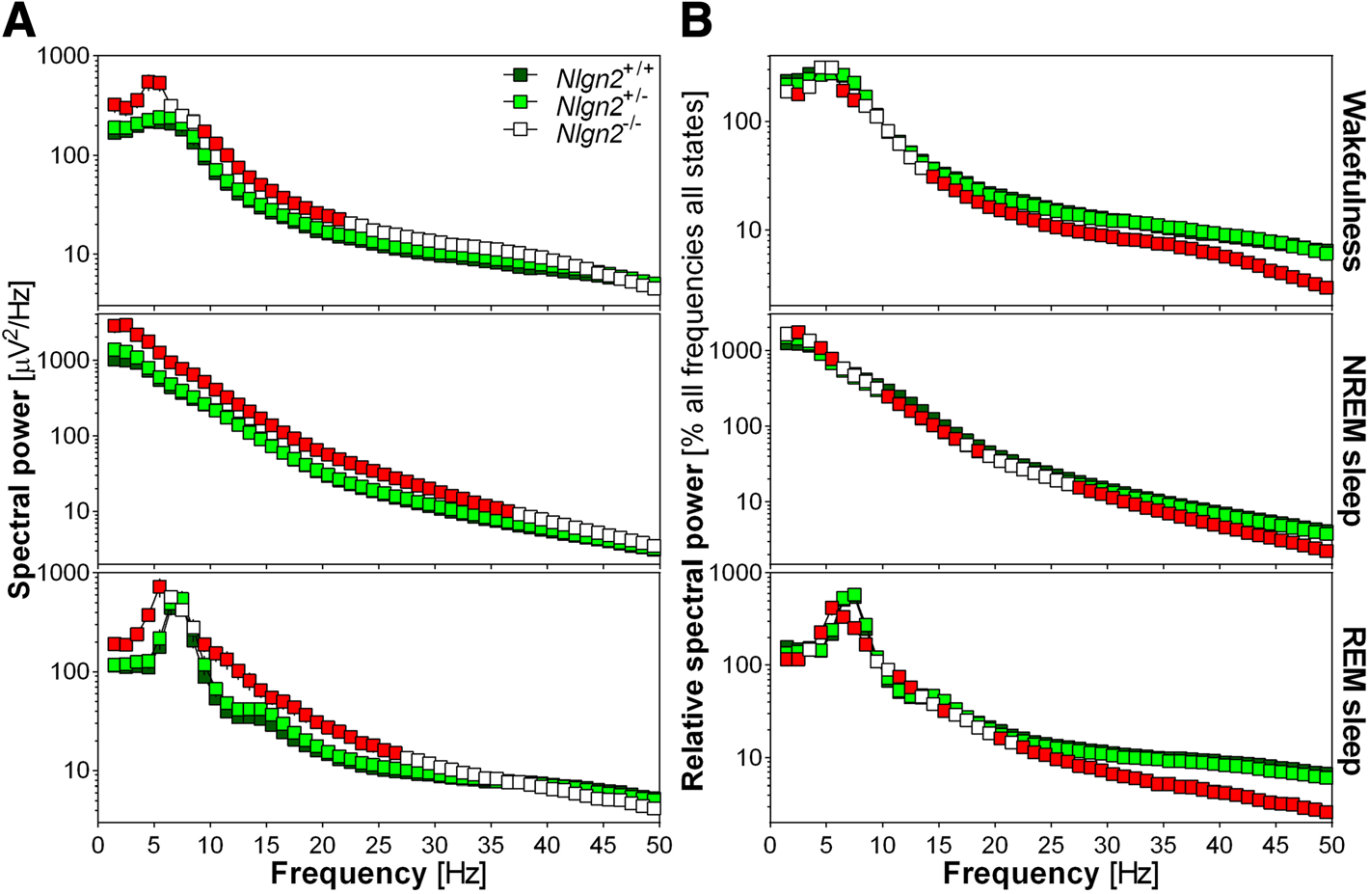
Power spectra of the 24 h EEG recording computed between 1 and 50 Hz with a 1-Hz resolution separately for the three vigilance states in *Nlgn2*^*+/+*^, *Nlgn2*^*+/−*^ and *Nlgn2*^*−/−*^ mice. **a**) Absolute spectral power for wakefulness, NREM sleep and REM sleep. For wakefulness, significant genotype effects were found for frequencies between 1 and 6 Hz and between 9 and 22 Hz (F_2,37_ > 3.9, *p* < 0.03). For NREM sleep, significant genotype effects were found for frequencies between 1 and 37 Hz (F_2,37_ > 3.4, p < 0.05). For REM sleep, significant genotype effects were found for frequencies between 1 and 6 Hz and between 9 and 27 Hz (F_2,37_ > 3.2, p < 0.05). **b**) Relative spectral power for wakefulness, NREM sleep and REM sleep. For wakefulness, significant genotype effects were found for frequencies 2 to 3 Hz, 6 to 8 Hz, and 14 to 50 Hz (F_2,37_ > 3.6, *p* < 0.04). For NREM sleep, significant genotype effects were found for frequencies 2 to 3 Hz, 4 to 6 Hz, 9 to 19 Hz, and 27 to 50 Hz (F_2,37_ > 3.3, p < 0.05). For REM sleep, significant genotype effects were found for frequencies 1 to 3 Hz, 4 to 9 Hz, 11 to 13 Hz, 15 to 16 Hz, 20 to 21 Hz, and 22 to 50 Hz (F_2,37_ > 3.3, p < 0.05). Red symbols indicate Hz-bins for which *Nlgn2*^*−/−*^ mice are significantly different from *Nlgn2*^*+/+*^ mice (simple effect analysis; p < 0.05). For clarity, significant differences between *Nlgn2*^*+/−*^ and *Nlgn2*^*+/+*^ mice have not been represented

Spectral analysis of the EEG revealed extensive alterations in spectral activity in all three vigilance states in *Nlgn2*^*−/−*^ mice. First, absolute spectral power was higher in *Nlgn2*^*−/−*^ mice than in *Nlgn2*^*+/+*^ mice for most frequencies below 23 Hz during wakefulness, below 38 Hz during NREM sleep and below 28 Hz during REM sleep (Fig. 4a). In general, vigilance state power spectra in *Nlgn2*^*+/−*^ mice were very similar to *Nlgn2*^*+/+*^ mice for the absolute computation. The global increase in absolute power in *Nlgn2*^*−/−*^ mice compared to *Nlgn2*^*+/+*^ mice could be the result of differences in the general organization of the cerebral cortex that would impact EEG activity in all states.

Second, in order to more adequately capture state-specific genotype differences, spectral power was expressed relative to the individual mean of all frequencies of all states. This revealed a different pattern of genotype differences where wakefulness spectral activity was lower in *Nlgn2*^*−/−*^ mice than in *Nlgn2*^*+/+*^ mice for some Hz-bins below 8 Hz and all Hz-bins above 14 Hz; NREM sleep spectral activity was higher in *Nlgn2*^*−/−*^ mice than in *Nlgn2*^*+/+*^ mice for some Hz-bins below 6 Hz, but lower in *Nlgn2*^*−/−*^ than in *Nlgn2*^*+/+*^ mice for most Hz-bins between 10 and 19 Hz and all bins above 27 Hz; REM sleep spectral activity was higher in *Nlgn2*^*−/−*^ than *Nlgn2*^*+/+*^ mice for frequencies 4–6 Hz and 11–13 Hz, but lower in *Nlgn2*^*−/−*^ than *Nlgn2*^*+/+*^ mice for frequencies 1–3 Hz, 6–9 Hz, 15–16 Hz, and most Hz-bins above 20 Hz (Fig. 4b). Also in the case of relative computation, vigilance state power spectra in *Nlgn2*^*+/−*^ mice were very similar to *Nlgn2*^*+/+*^ mice.

Finally, the peak of absolute theta activity during both wakefulness and REM sleep appeared to locate at slower frequencies in *Nlgn2*^*−/−*^ mice compared to littermates (i.e., at around 5 Hz in *Nlgn2*^*−/−*^ mice and around 6 Hz in *Nlgn2*^*+/+*^ mice for wakefulness; at around 5.5 Hz in *Nlgn2*^*−/−*^ mice and around 7.5 Hz in *Nlgn2*^*+/+*^ mice for REM sleep; Fig. 4a). However, only a difference in theta peak activity seemed to remain for REM sleep when relative power spectra were considered (5 Hz in *Nlgn2*^*−/−*^ mice and *Nlgn2*^*+/+*^ mice for wakefulness; 5.5 Hz in *Nlgn2*^*−/−*^ mice and 7.5 Hz in *Nlgn2*^*+/+*^ mice for REM sleep; Fig. 4b).

### Slower dynamics of NREM sleep delta activity in *Nlgn2*^*−/−*^ mice

**Fig. 5:**
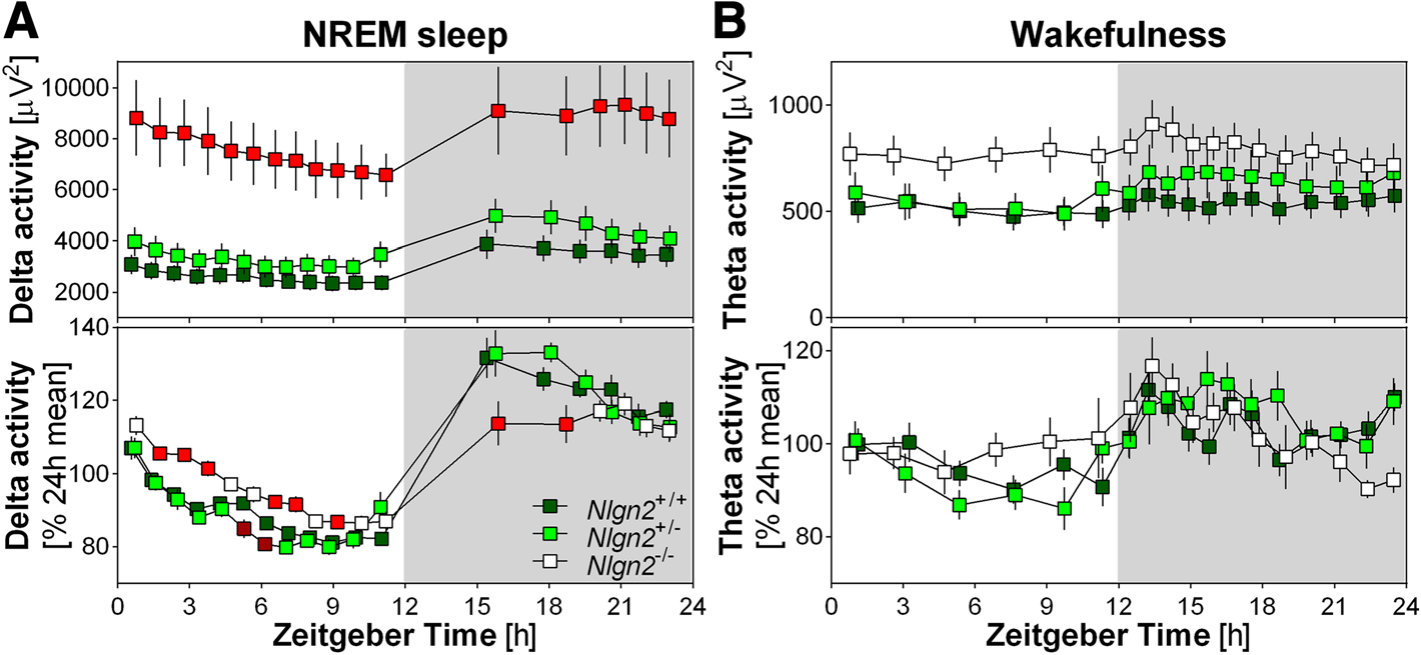
Twenty-four hour time course of delta activity (1–4 Hz) during NREM sleep and theta activity (6–9 Hz) during wakefulness in *Nlgn2*^*+/+*^, *Nlgn2*^*+/−*^ and *Nlgn2*^*−/−*^ mice measured using EEG. **a**) 24 h dynamics of NREM sleep absolute delta activity (upper panel) and relative delta activity (lower panel). For absolute activity, significant genotype and interval effects have been found (respectively, F_2,37_ = 11.7, p < 0.001 and F_17,629_ = 38.0, *p* < 0.0001), as well as a tendency for significant interaction (F_34,629_ = 2.3, *p* = 0.05). For relative activity, a significant interaction has been observed (F_34,629_ = 3.4, p < 0.0001). Red symbols indicate intervals for which *Nlgn2*^*−/−*^ mice are significantly different from *Nlgn2*^*+/+*^ mice, and dark red symbols indicate intervals for which *Nlgn2*^*+/−*^ mice are significantly different from *Nlgn2*^*+/+*^ mice (simple effect analysis; p < 0.05). **b**) 24 h dynamics of waking absolute theta activity (upper panel) and relative theta activity (lower panel). For both absolute and relative activity, a significant interval effect was found (respectively, F_17,612_ = 3.6, p < 0.01 and F_17,612_ = 5.3, p < 0.0001), but no significant genotype effect (respectively, F_2,36_ = 2.2, *p* = 0.13 and F_2,36_ = 0.7, *p* = 0.5) or interaction (respectively, F_34,612_ = 1.2, *p* = 0.2 and F_34,612_ = 1.3, p = 0.13) was observed. Grey backgrounds indicate the 12 h dark period

The time course of NREM sleep delta activity was also compared between genotypes using absolute and relative power calculations (Fig. 5a). Absolute delta activity was significantly and globally higher in *Nlgn2*^*−/−*^ mice compared to *Nlgn2*^*+/+*^ mice (more than two times higher as also observed in Fig. 4a), but the 24-h dynamics only showed a tendency to be affected by genotype. However, a between-genotype difference in the 24-h dynamics was revealed by analyzing the relative delta activity time course, which showed higher delta in *Nlgn2*^*−/−*^ mice compared to *Nlgn2*^*+/+*^ mice for many intervals during the light period and lower delta in *Nlgn2*^*−/−*^ than in *Nlgn2*^*+/+*^ mice for the first two intervals of the dark period. Given that *Nlgn2*^*−/−*^ mice showed preserved NREM sleep duration during their main rest period (i.e., 12-h Light; Fig. 1b) and unaltered delta activity level at the beginning and end of the rest period, this observation suggests a slower dissipation of delta activity during the light (rest) period as well as a slower build-up of delta activity during the dark (active) period in *Nlgn2*^*−/−*^ mice.

A recent study proposed that it is specifically wakefulness dominated by theta (6–9.5 Hz) activity that drives homeostatic build-up of NREM sleep delta activity [33]. Accordingly, we investigated the 24-h time course of both absolute and relative theta activity during wakefulness in *Nlgn2*^*−/−*^ mice and littermates (Fig. 5b). Although significant daily variations in both absolute and relative theta activity were found, both the dynamics and the global theta activity level were not significantly affected by genotype.

## Discussion

We here report various alterations in wakefulness and sleep quantity and quality in mice lacking NLGN2. More precisely, *Nlgn2*^*−/−*^ mice showed an increased duration of wakefulness and decreased durations of sleep states, with changes in wakefulness and NREM sleep attributable to the active (dark) period, and those in REM sleep found for the full nychthemeron. In addition, *Nlgn2*^*−/−*^ mice exhibited a general increase in the consolidation of wakefulness and NREM sleep as well as abnormal EEG bursts of high amplitude predominately during wakefulness and REM sleep. Vigilance state quality was further affected in mice lacking NLGN2 as indexed with widespread changes in EEG activity in all states, and indications of dampened dynamics of sleep homeostasis (i.e., time course of NREM sleep delta activity). These observations point to different roles of NLGN2 in sleep regulation, and suggest that multiple sleep regulatory systems are impacted by the absence of NLGN2.

The first hypothesis of the current study was that the absence of NLGN2 would result in more wakefulness and less sleep, which is supported by the results. This assumption was based on the flip-flop switch model of sleep regulation [19, 34], supposing that, in the absence of NLGN2, GABAergic outputs from sleep-inducing regions such as the hypothalamic ventrolateral preoptic nucleus (VLPO) and extended VLPO are reduced, thus favoring wakefulness. In this model, inhibitory outputs of sleep-inducing regions are notably targeting orexin/hypocretin neurons of the lateral hypothalamus, a cell population on which NLGN2 was specifically shown to be increased under high sleep need (i.e., after sleep deprivation) [35]. Given the role of NLGN2 in promoting inhibitory transmission [16] and the role of orexin/hypocretin neurons in promoting transitions to wake [36], upregulation of NLGN2 could favor inhibition of orexin/hypocretin neurons and sleep. In *Nlgn2*^*−/−*^ mice, a global reduction of the inhibition on orexin/hypocretin neurons would favor wake. Furthermore, a role for NLGN2 in orexin/hypocretin neurons could represent a mechanism by which alterations in wakefulness and NREM sleep consolidation occur in *Nlgn2*^*−/−*^ mice, because pharmacological and genetic manipulations of the orexin/hypocretin system have been shown to impact variables linked to wake/sleep consolidation/fragmentation in rodents [33, 37].

In parallel to the orexin/hypocretin system, other GABAergic innervated regions could also be implicated in the wakefulness/sleep phenotype observed in the absence of NLGN2. For instance, GABAergic neurons from the medullary parafacial zone were shown to promote NREM sleep in mice likely via projections to the parabrachial nucleus [38]. An absence of NLGN2 in parabrachial neurons receiving parafacial GABA projections may thus alleviate inhibitory transmission and decrease NREM sleep. In addition, a subtype of GABAergic neurons (i.e., expressing Lhx6) of the zona incerta was recently shown to promote both NREM and REM sleep [39]. *Nlgn2*^*−/−*^ mice could thus express less sleep via impairment in GABAergic transmission in areas targeted by this specific cell population. Additional studies aiming at quantifying NLGN2 presence in these defined brain regions and eventually assessing sleep variables under downregulation of NLGN2 in some of these regions will further the understanding of mechanisms behind the role of NLGN2 in regulating wakefulness and sleep duration.

The second hypothesis of this study stated that the absence of NLGN2 would modify EEG activity because NLGN2 regulates GABA_A_ receptors [17, 23], which are shaping EEG activity [20]. Our observations of hypersynchronized EEG events during wakefulness and REM sleep as well as of multiple alterations in EEG activity in all vigilance states support this hypothesis. The report of increased excitability under NLGN2 downregulation [17] is consistent with the implication of NLGN2 in inhibitory synaptic transmission [15, 16], and may suggest a role for NLGN2 in epileptogenesis. Such a role could also be supported by our observation of abnormal ‘hypersynchronized’ EEG events in *Nlgn2*^*−/−*^ mice. Interestingly, very similar ‘hypersynchronized’ EEG events, which were considered as seizure spiking activity, have also been observed under overexpression of *Nlgn2* in the forebrain [15]. Indeed, this study found spiking events of, on average, 7.4 Hz and 1.7 s that predominated during wakefulness and REM sleep [15]. Together with this study, our findings suggest that a delicate balance of NLGN2 is required to prevent ‘hypersynchronized’, potentially epileptiform, EEG activity. Of note is that a predominance of seizures during wakefulness and REM sleep has been reported for another mouse model showing increased wakefulness amount (i.e., *Kcna2* KO mice) [40]. The predominance of abnormal EEG events during wakefulness and REM sleep could point to a role for NLGN2 in cholinergic transmission, given that cholinergic tone is high specifically during these two vigilance states [19]. In fact, NLGN2 has been reported to localize at synapses from cholinergic cells in multiple areas of the mouse brain (e.g., hippocampus, somatosensory and medial prefrontal cortex) [41]. Cholinergic activity is well-known to shape activity and responsiveness of neurons of the cerebral cortex [42], and a role for NLGN2 in the regulation of cholinergic outputs could thus also explain the widespread changes in EEG activity observed in *Nlgn2*^*−/−*^ mice in the present study. Accordingly, future investigations should assess the role of NLGN2 in cholinergic neurotransmission and the contribution of this specific role to EEG activity.

Alterations of GABAergic neurotransmission in thalamocortical circuits are also likely to contribute to modifications of EEG activity during wakefulness, NREM and REM sleep in *Nlgn2*^*−/−*^ mice. The general increase in absolute spectral activity, especially for frequencies overlapping alpha, sigma and beta activity bands (i.e., 9 to 22 Hz) could originate from a global deficit in inhibition as pointed out by decreased mIPSCs and increased excitability reported under *Nlgn2* downregulation [16, 17], and by the role of NLGN2 in the development of GABAergic synapses [43]. In parallel, our observation of high delta activity in *Nlgn2*^*−/−*^ mice is consistent with findings of decreased spectral power in the delta range under administration of GABA_A_ receptor agonists [21, 22, 44], given the regulation of the clustering of GABA_A_ receptors by NLGN2 [23]. Of interest is that even if *Nlgn2*^*−/−*^ mice express higher absolute delta power during NREM sleep compared to littermates, they show a slower daytime dynamics of delta activity as observed using relative quantification. Indeed, the decay of relative delta activity during the light phase is more gradual, apparently following a linear instead of an exponential trend, and the build-up of delta activity during the dark phase is also less abrupt. According to the two-process model of sleep regulation [29], such a time course of delta activity may suggest a slower dynamics of homeostatic sleep pressure. Furthermore, a slower build-up of homeostatic sleep pressure (or need) may represent another explanation for an increase capacity to stay awake and a reduced NREM sleep duration observed in absence of NLGN2.

Here, we have assessed the dynamics of the main sleep homeostasis marker (i.e., delta activity) in absence of NLGN2 under normal/undisturbed conditions, and found indications of slower dynamics of both decay and build-up as detailed above. Although a sleep homeostasis phenotype could be more evident under baseline/undisturbed conditions for some genetic models, as shown for *Orexin*/*Hypocretin* KO mice [33], an altered dynamics of sleep need could rather be more apparent under challenged conditions such as enforced wakefulness. For instance, in *Nlgn1* KO mice, sleep deprivation specifically revealed a major exacerbation of the delta activity rebound indicative of a faster build-up of sleep need in absence of NLGN1 [10]. A next step in understanding the role of NLGN2 in sleep regulation could thus be to monitor recovery sleep after sleep deprivation in mice with modifications of *Nlgn2* expression.

The full KO model studied in the current study has allowed to verify the implication of NLGN2 in the regulation of sleep quantity and quality, but sleep duration and EEG alterations observed after total gene ablation could be attributable to developmental defects and/or compensations [45]. To avoid such confounding effects, in addition to investigating the precise brain and cellular locations mediating the roles of NLGN2, future research will need to downregulate *Nlgn2* expression in specific brain areas and cell types at given developmental ages using, for instance, viral strategies or conditional gene KO techniques. Nonetheless, the present findings have the value of justifying such additional, more targeted, investigations.

Sleep disorders have been considered as a serious economic burden because people suffering from sleep disorders utilize more medical resources and have higher risks of developing comorbid medical or psychiatric disorders [46, 47]. Understanding mechanisms underlying the role of NLGN2 in sleep regulation will uncover, at least to some extent and mainly for neuropsychiatric conditions, the etiology of comorbidity as well as provide potential therapeutic targets for comorbid disorders. Our findings support roles of NLGN2 in the regulation of multiple sleep dimensions and encourage future investigations aimed at unravelling the cellular and molecular mechanisms behind.

## Abbreviations

ANOVA: Analysis of variance
ASDs: Autism spectrum disorders
E/I: Excitation to inhibition
EEG: Electroencephalographic
EMG: Electromyographic
GABA: γ-aminobutyric acid
Hz: Hertz
KO: Knockout
mIPSCs: Miniature inhibitory postsynaptic currents
NLGN: Neuroligin
NMDA: *N*-methyl-D-aspartate
NREM: Non-rapid eye movement
REM: Rapid eye movement
SEM: Standard error of the mean
VLPO: Ventrolateral preoptic nucleus of the hypothalamus
ZT: Zeitgeber time
