# Correction to: The effect of Neuroligin-2 absence on sleep architecture and electroencephalographic activity in mice

**DOI:** 10.1186/s13041-019-0425-8

**Published:** 2019-01-30

**Authors:** Bong Soo Seok, Feng Cao, Erika Bélanger-Nelson, Chloé Provost, Steve Gibbs, Zhengping Jia, Valérie Mongrain

**Affiliations:** 1 Research Center and Center for Advanced Research in Sleep Medicine, Hôpital du Sacré-Cœur de Montréal (CIUSSS-NIM), 5400 Gouin West blvd, Montréal, QC H4J 1C5 Canada; 2 Department of Neuroscience, Université de Montréal, 2960 chemin de la Tour, Montreal, QC H3T 1J4 Canada; 3 Neurosciences and Mental Health, The Hospital for Sick Children, Toronto, ON M5G 1X8 Canada; 4 Department of Physiology, University of Toronto, Toronto, ON M5S 1A8 Canada

**Correction to:** ***Molecular Brain*** **(2018) 11:52**

**https://doi.org/10.1186/s13041-018-0394-3**

Following publication of the original article [1], the authors reported that the article was mistakenly submitted with the omission of two authors: Feng Cao and Zhengping Jia. The authors declare that this was an error made in good faith. The corrected author list and list of affiliations are used in this Correction. The changes made to the author list and list of affiliations are also listed below, as well as the revised ‘Acknowledgements’ section and ‘Authors’ contributions’ section.

- Feng Cao has been added to the author list between Bong Soo Seok and Erika Bélanger-Nelson with affiliations number 3 (Neurosciences and Mental Health, The Hospital for Sick Children, Toronto, ON M5G 1X8, Canada) and number 4 (Department of Physiology, University of Toronto, Toronto, ON M5S 1A8, Canada).
- The † symbol has been added to Bong Soo Seok and Feng Cao, to indicate that these authors have contributed equally to this work.
- Zhengping Jia has been added to the author list between Steve Gibbs and Valérie Mongrain with affiliations number 3 (Neurosciences and Mental Health, The Hospital for Sick Children, Toronto, ON M5G 1X8, Canada) and number 4 (Department of Physiology, University of Toronto, Toronto, ON M5S 1A8, Canada).
- Zhengping Jia should be removed from the ‘Acknowledgements’ section as this person is a co-auhtor. The revised ‘Acknowledgements’ section consequently is: “The authors thank Cassandra Areal who helped with EEG scoring and analysis, colleagues who helped with SD (Julien Dufort-Gervais, Lydia Hannou, Marlène Freyburger) and Gaétan Poirier for technical help.”
- The sentence “FC and ZJ designed some analyses” should be added to the ‘Authors’ contributions’ section. The revised ‘Authors’ contributions’ section consequently is: “BSS analyzed and interpreted the data, and wrote the paper. EBN and CP performed the experiments. FC and ZJ designed some analyses. SG participated in data interpretation. VM designed the experiment, analyzed and interpreted the data, and wrote the paper. All authors read and approved the final manuscript.”
